# Transient Giant R Wave as a Marker for Ischemia in Unstable Angina

**DOI:** 10.7759/cureus.1200

**Published:** 2017-04-28

**Authors:** Yashasvi Chugh, Carola Maraboto, Panagiota Christia, Robert Faillace

**Affiliations:** 1 Internal Medicine, Jacobi Medical Center; 2 Medicine, Jacobi Medical Center; 3 Cardiology, Jacobi Medical Center

**Keywords:** giant r wave, electrocardiography, unstable angina

## Abstract

Unstable angina is a clinical diagnosis that may present with or without electrocardiographic changes. The “giant R wave” on electrocardiogram has been reported as a manifestation of acute ischemia; however, it is a rare finding in current clinical practice. We describe a case of a patient with unstable angina and a transient “giant R wave” pattern with a culprit lesion in the right coronary artery.

## Introduction

Unstable angina (UA) and non-ST elevation myocardial infarction (NSTEMI) comprise the so-called non-ST elevation acute coronary syndromes (NSTE-ACS). The diagnosis of UA is made on clinical grounds and it is considered to be present in patients with ischemic symptoms suggestive of an acute coronary syndrome, with or without changes indicative of ischemia on the body surface 12-lead electrocardiogram (ECG), such as ST segment depression, transient ST elevation, or T wave inversion. This differs from NSTEMI mainly in the degree of ischemia since, in such case, there is enough myocardial damage to detect elevation of cardiac biomarkers of necrosis [1].

Other electrocardiographic changes during myocardial ischemia have been reported, but are rare and not widely described in the literature. The presence of “giant R waves,” defined as the development of a 50% or greater increase in R wave amplitude during myocardial ischemia, was first reported by Rakita and colleagues in 1954 [2] after it was observed following occlusion of a coronary artery in dogs. We report a case of UA presenting with a transient “giant R wave” of acute myocardial ischemia in lead V2 that was found to have a culprit lesion in the right coronary artery (RCA).

## Case presentation

A 55-year-old man presented to our emergency department with multiple episodes of mild sub-sternal chest discomfort, burning in nature, with associated numbness of his left arm. The patient had a history of polymyositis, interstitial lung disease and ischemic cardiomyopathy with a recent NSTEMI for which he underwent percutaneous coronary intervention (PCI) of the left anterior descending artery (LAD) with placement of drug eluting stent six weeks prior to admission to the hospital.

**Figure 1:**
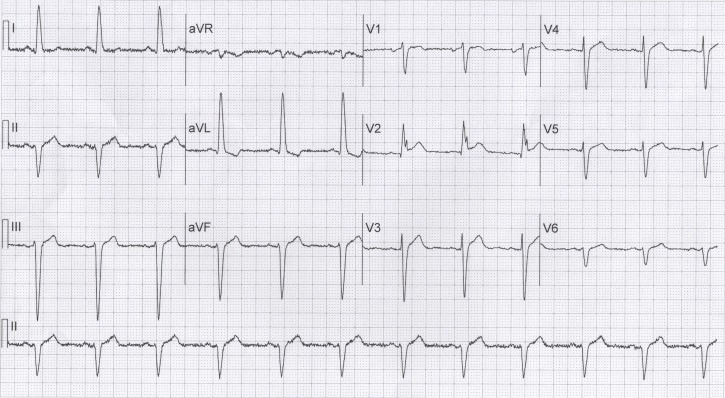
ECG on admission showing 'Rsr’ pattern in V2 (“giant R wave”)

**Figure 2:**
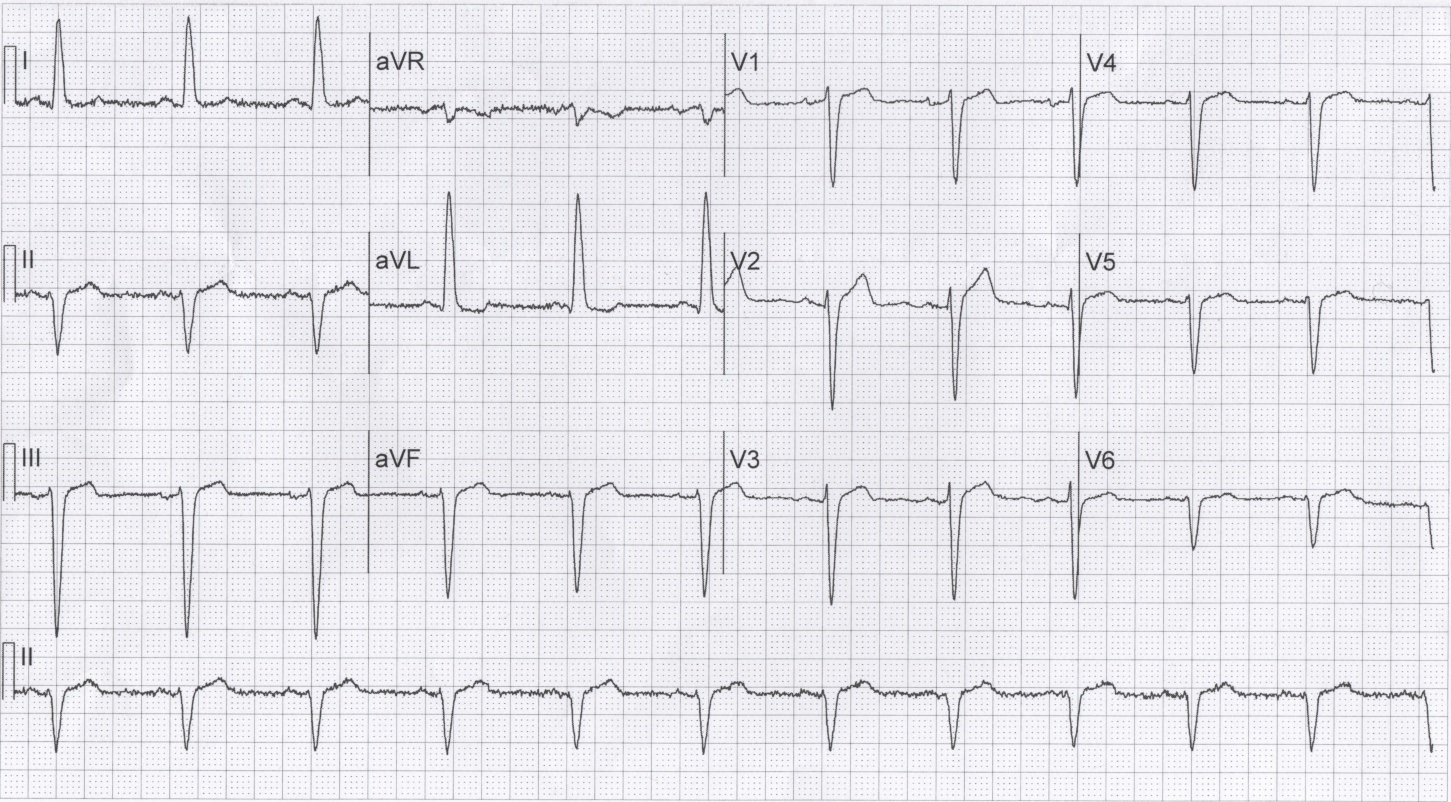
Repeat ECG after 14 hours demonstrating resolution of QRS changes in V2

Further evaluation revealed a normal cardiac exam; however, an initial ECG in the setting of chest discomfort demonstrated normal sinus rhythm, left axis deviation, left anterior fascicular block and a 2 mm concave ST segment elevation along with findings consistent with a giant R wave and a Rsr’ pattern in lead V2 (Figure 1). Given the initial presentation, a diagnosis of NSTE-ACS was made and medical treatment for this was initiated. Cardiac enzymes and liver function tests were normal. The patient’s ECG, 14 hours later (Figure 2), in the absence of chest discomfort, revealed resolution of the giant R wave morphology. The patient was diagnosed with unstable angina and he was sent for coronary angiography, which revealed a patent proximal LAD stent, mid-LAD with 50% stenosis, distal LAD with 75% stenosis, first diagonal with 90% stenosis, first obtuse marginal with 40% stenosis, and mid-RCA with 75% stenosis and a type C lesion that was thought to be the culprit lesion. He underwent PCI with placement of a drug eluting stent to the RCA and, after 48 hours, the patient was discharged home in stable condition.

## Discussion

The “giant R wave” of acute myocardial ischemia has previously been reported in patients with acute myocardial infarction [3-5], in variant (Prinzmetal) angina [6], during exercise treadmill stress test [7-8], in the setting of percutaneous transluminal coronary angioplasty [9], and following coronary artery ligation in dogs [2]. Giant R waves, to our knowledge, have not yet been reported in the setting of unstable angina. Severe myocardial ischemia from total coronary occlusion can also lead to ST segment elevation, widening of the QRS complex and obliteration of the S wave [7, 10], constituting the “giant R wave pattern”. This composite is usually evident in the hyperacute phase of ischemia, which is the reason why this has been a rare finding. However, advances in treatment of acute coronary syndromes with focus on immediate intervention have allowed physicians to witness early stages of ischemia and detect these changes.

The electrophysiological mechanism underlying the “giant R waves” is not well understood, but it is believed to be secondary to an abnormal depolarization of an ischemic myocardial region. One theory suggests that severe myocardial ischemia leads to the release of intracellular potassium from cardiomyocytes, leading to a local intra-myocardial conduction delay with concomitant extracellular positive electrical gradient in the affected area while the rest of the heart is in repolarization, with a resultant unopposed accentuated and broad R wave in leads facing the area of acute severe ischemia [4-5, 7]. The changes in the terminal portion of the QRS complex are a consequence of the conduction abnormalities at the level of the Purkinje fibers, which normally develop only after a severe and prolonged episode of ischemia [10].

## Conclusions

The presence of a “giant R wave” pattern in the ECG of a patient reporting ischemic symptoms should support the diagnosis of acute coronary syndrome, primarily indicating that the patient is undergoing the hyperacute phase of ischemia and should prompt early intervention. As mentioned above, changes in the QRS complex usually occur in leads facing zones of myocardial ischemia; we speculate that this phenomenon was occurring in the subendocardial region of our patient’s left ventricular posterior wall, leading to transiently occurring giant R waves associated with an 'Rsr’ pattern and ST segment elevation in lead V2.

## References

[1] Amsterdam EA, Wenger NK, Brindis RG (2014). 2014 AHA/ACC Guideline for the management of patients with non–ST-elevation acute coronary syndromes, a report of the American College of Cardiology/American Heart Association Task Force on practice guidelines. Circulation.

[2] Rakita L, Borduas JL, Rothman S (1954). Studies on the mechanism of ventricular activity. XII. Early changes in the RS-T segment and QRS complex following acute coronary artery occlusion: experimental study and clinical applications. Am Heart J.

[3] Madias JE (1977). The earliest electrocardiographic sign of acute transmural myocardial infarction. J Electrocardiol.

[4] Faillace RT, Akiyama T, Chang W (1985). The giant R wave of acute myocardial infarction. Jpn Heart J.

[5] Madias JE, Attari M, Bravidis D (2001). Giant R-waves in a patient with an acute inferior myocardial infarction. J Electrocardiol.

[6] Prinzmetal M, Ekmekci A, Kennamer R (1960). Variant form of angina pectoris, previously undelineated syndrome. JAMA.

[7] Ortega-Carnicer J (2004). Giant R wave, convex ST-segment elevation, and negative T wave during exercise treadmill test. J Electrocardiol.

[8] Testa-Fernandez A, Rios-Vazquez R, Sieira-Rodriguez-Moret J (2011). "Giant R wave" electrocardiogram pattern during exercise treadmill test: a case report. J Med Case Rep.

[9] Wagner NB, Sevilla DC, Krucoff MW (1988). Transient alterations of the QRS complex and ST segment during percutaneous transluminal balloon angioplasty of the left anterior descending coronary artery. Am J Cardiol.

[10] Birnbaum Y, Drew BJ (2003). The electrocardiogram in ST elevation acute myocardial infarction: correlation with coronary anatomy and prognosis. Postgrad Med J.

